# Insights Into Provider Bias in Family Planning from a Novel Shared Decision Making Based Counseling Initiative in Rural, Indigenous Guatemala

**DOI:** 10.9745/GHSP-D-19-00377

**Published:** 2020-03-30

**Authors:** Meghna Nandi, Jillian Moore, Marcela Colom, Andrea del Rosario Garcia Quezada, Anita Chary, Kirsten Austad

**Affiliations:** a Warren Alpert Medical School, Brown University, Providence, RI, USA.; b Wuqu' Kawoq | Maya Health Alliance, Bethel, VT, USA.; c Department of Family and Community Medicine, University of New Mexico, Albuquerque, NM, USA.; d Department of Emergency Medicine, Massachusetts General Hospital, Boston, MA, USA.; e Department of Family Medicine, Boston University School of Medicine, Boston Medical Center, Boston, MA, USA.

## Abstract

Race, ethnicity, and indigenous status should be considered as potential drivers of provider bias in family planning services globally. Efforts to confront provider bias in family planning counseling should include concrete strategies that promote provider recognition of biases and longitudinal curriculums that allow for sustained feedback and self-reflection.

See related article in Solo and Festin.

Resumen en español al final del artículo.

## INTRODUCTION

An article by Solo and Festin^1^ discusses the importance of addressing provider bias in family planning services. We agree that provider bias in family planning services is a widespread problem that restricts clients' autonomy and empowerment and applaud the authors for directing a spotlight on this important issue.

Our goals in writing this response are 2-fold. First, drawing from our experiences providing family planning services to primarily indigenous Maya women in rural Guatemala, we would like to expand Solo and Festin's discussion on bias against specific groups to include race and ethnicity. In this article, we understand race is defined as a social group based on perceived skin color or other physical qualities and ethnicity is defined as a social group based on common cultural or national traditions.^2^

Solo and Festin's article highlights sources of client-based bias, including age, parity, and marital status, as well as biases against specific socially marginalized groups, emphasizing youth, women who have HIV, women seeking abortion, those with disabilities, and men seeking permanent contraception. However, race and ethnicity are not singled out as a specific source of bias in their article. In our family planning work, ethnic minority patients report judgment, bias, and coercion in their reproductive health care experiences. We hope to use our professional observations and the current literature on racial and ethnic biases in health care to build on Solo and Festin's article by including race and ethnicity as factors that merit recognition in this larger discussion of provider bias in family planning.

Second, we would like to complement Solo and Festin's discussion about how to address provider bias of all types by sharing specific strategies we have used in our work. After years of providing family planning services in rural Guatemala, we have seen how training that does not directly confront bias has limited power to promote quality counseling rooted in client autonomy and choice. Here, we hope to share insight from our own on-the-ground efforts to eliminate provider bias in our family planning program.

We hope to share insight from our own on-the-ground efforts to eliminate provider bias in our family planning program.

## OUR CONTEXT

We have been involved in women's health programs at the nongovernmental organization (NGO) Wuqu' Kawoq | Maya Health Alliance, which was founded to address a lack of culturally and linguistically appropriate health and social services for indigenous people in Guatemala. Nearly half of Guatemalans are of indigenous Maya descent^3^ and have sociocultural practices, such as speaking indigenous languages and wearing traditional clothing, that distinguish them from those of European or mixed ancestry. Although Guatemala has recently been reclassified from a lower- to a middle-income country, most Maya citizens live on less than US$1 per day. Limited access to quality health care due to language barriers, cultural differences, and widespread discrimination^4^ perpetuates these inequalities. The Guatemalan constitution guarantees free health care to all its citizens, but the public health system is underfunded and has been unable to provide adequate care to rural indigenous areas of the country.^5^^,^^6^

Indigenous women in rural areas have a higher unmet need for modern contraception than their nonindigenous counterparts (43.4% vs. 26.7% according to most recent estimates).^3^^,^^7^^–^^9^ The public sector is the largest source of family planning services in Guatemala and offers women a range of methods for free including female surgical sterilization, oral contraceptive pills, condoms, copper intrauterine devices, injectables, and implants.^3^^,^^10^ The quality of family planning services is questionable. Public clinics have frequent shortages of contraceptive methods.^10^ In our clients' experiences, long-acting reversible contraceptive (LARC) method placement and removal were not offered daily but rather were provided through intermittent missions coordinated by health centers. Strikingly, the majority of public sector providers are not indigenous and do not speak local Mayan languages,^6^ even though the public sector is often the most affordable and geographically accessible health care option for rural indigenous communities. Nearly all visits are conducted in Spanish,^3^ which further increases barriers to care for monolingual speakers^5^^,^^6^^,^^11^ and may contribute to disparities in reproductive health care utilization and contraceptive use between indigenous and nonindigenous women.^9^

A number of NGOs have attempted to fill in these gaps in family planning services. NGOs provide women with the same methods available in the public sector for a nominal cost, often determined by her capacity to pay, or free of charge (as in the case of Wuqu' Kawoq).^10^^,^^12^^,^^13^ Asociación Pro-bienestar de la Familia, affiliated with the International Planned Parenthood Fund, is the largest NGO providing these services and one of the largest family planning providers nationally with approximately 25 health centers spanning the country.^10^^,^^12^ Although private hospitals, clinics, and pharmacies also offer contraceptive services, their fees are prohibitive for most Maya women.^3^^,^^10^

## PROVIDER BIAS BASED ON RACE AND ETHNICITY

Higher unmet need for contraception among indigenous Guatemalans is likely multifactorial. Structural inequalities faced by Maya women, including poverty, rural isolation, and language barriers, are well-documented and are at least partly to blame for reproductive health disparities.^11^ Provider bias based on age and parity—among those highlighted by Solo and Festin—may also play a role as Maya women begin childbearing earlier and have larger family sizes than the general Guatemalan population. In addition, our experiences as health care providers suggest discrimination based on race/ethnicity as an important contributing factor.

There is sound empirical evidence of racial and ethnic biases among providers generally in health care broadly^14^^–^^16^ and specifically in family planning care. Most studies to date have been conducted in the United States. For example, a 2008 study found that black women were more likely to report having felt pressured by a provider to use a particular contraceptive method than white women.^17^ Similarly, a national patient survey found that minority women in the United States were more likely to be counseled on birth control including sterilization.^18^

Other research has sought to directly measure providers' racial and ethnic bias, either explicitly through self-report of conscious attitudes or implicitly. For example, 2 studies presented providers with clinical scenarios involving patients identical apart from race and found that health care providers were more likely to recommend LARCs or sterilization to minority patients than to white patients.^19^^,^^20^ Implicit bias in medical providers has also been assessed using the implicit association test, which is supported by strong psychometric evidence. The implicit association test serves as a proxy for attitudes that people are unwilling or unable to report because these perceptions are unconscious and are a predictor of discriminatory behavior.^21^ Multiple systematic reviews have confirmed that implicit racial bias exists among health care professionals at the same rates as the general population, though to our knowledge none has focused explicitly on family planning providers. It is worth highlighting that racial and ethnic bias is more likely to manifest in areas like family planning in which decisions depend strongly on patient preference and thus demand personalized counseling. One study in the field of genetic counseling, similarly directed by patient preference, found that higher pro-white implicit bias was associated with less individualized counseling of minority patients.^22^

Racial and ethnic bias is more likely to manifest in areas like family planning in which decisions depend strongly on patient preference and demand personalized counseling.

Racial and ethnic bias in family planning deserves special attention for multiple reasons. First and foremost, race and ethnic groups are not biologic entities but instead social constructs. Despite abundant scientific inquiry regarding a biologic basis for race, compelling evidence is lacking. As such, there is no evidence to support offering disparate contraceptive recommendations by race. Compare this to other biases such as parity, where biologic differences such as the increased risk for obstetric complications in grand multiparity could justify making unique recommendations based on this characteristic.

Second, racial and ethnic bias is more likely than other forms of bias to manifest implicitly. In many medical contexts it is not socially acceptable to express overtly racist views. However, implicit bias is not manifested through reported beliefs, but rather is detected by methods revealing subconscious beliefs, like the implicit association test, and through subtle behaviors, like poor communication. Well-intentioned providers may find it difficult to accept evidence that their behaviors contribute to racial and ethnic disparities in care, especially when this conflicts with their explicit beliefs and morals. Because it is implicit, racial and ethnic bias remains largely invisible, leading to the dangerous and false conclusion that “racism” has been erased from medicine.

Racial and ethnic bias remains largely invisible, leading to the dangerous and false conclusion that “racism” has been erased from medicine.

Third, the strength of the evidence demonstrating bias is more substantial for race and ethnicity than any other category covered in Solo and Festin's review. In fact, a recent systematic review of implicit bias included 42 studies, 27 of which examined race and ethnicity as compared to only 14 for gender and 11 for age.^23^

Fourth, the most egregious examples of bias in family planning—forced or coerced sterilization—is most commonly linked to race and ethnicity. In contrast, nearly all examples of bias provided by Solo and Festin are related to withholding contraception from women who desire it or limiting their range of choices. Those of us who are family planning providers in the United States are acutely aware of our not-so-distant history of forced sterilization against women of color, including thousands of American Indian women.^24^^,^^25^ Similarly, across the world, sterilization without informed consent has been documented well into modern day.^26^

## RACIAL AND ETHNIC BIAS IN LOW-RESOURCE SETTINGS

Stories from the patients we care for support the existence of ethnoracial bias in public sector family planning services in Guatemala as well. One indigenous client described to us how she had hoped to have more children, but the doctors at the public hospital “would not permit it” and badgered her in to signing the consent form for tubal ligation because she had already had 2 cesarean deliveries. Another indigenous woman reported how during her last birth, she was asked repeatedly by the nonindigenous doctors why she would not agree to surgical sterilization and interrogated about how much land and money she had to support her children. When our NGO started offering contraceptive implants, there was high uptake among indigenous women even in villages where women could also access them with no cost in public health centers. Many of these women reported that because they were repeatedly told in public health centers that they had “too many children” they feared the doctor would refuse to remove the implant if they were unsatisfied or desired pregnancy. One woman stated “they do not value our children because we are dark-skinned.” In other instances, women have expressed uncertainty about whether or not they were left sterile following their last cesarean delivery at the public hospital. Indeed, an ethnographic study of maternal health in a rural Guatemalan community documents women forcibly undergoing tubal ligations after cesarean delivery without giving consent.^27^

Our patients‘ anecdotal experiences of perceived discrimination are supported by empiric evidence. Numerous qualitative studies have documented the discrimination indigenous Guatemalans face in public health facilities that actively deters them from seeking care.^11^^,^^27^^–^^29^ Similar experiences are shared by the other 370 million indigenous people worldwide.^30^^–^^35^ Multiple studies have identified implicit bias among health care providers favoring white over indigenous ethnicities.^30^^–^^32^^,^^35^ One study also found an association between ethnic bias and clinical recommendations and beliefs about patient compliance.^35^ A study from New Zealand found that indigenous patients were more likely to be started on a riskier form of dialysis treatment than their nonindigenous counterparts, even when controlling for socioeconomic factors and clinical comorbidities.^33^ In a qualitative study, health care providers in Canada also described discrimination toward indigenous patients affecting clinical practice.^34^

Patients' anecdotal experiences of perceived discrimination are supported by empiric evidence.

However, there is a relative paucity of research examining racial and ethnic biases of family planning health workers in low-resource settings. One study interviewed 108 family planning providers in public clinics in rural areas of Guatemala.^36^ More than half reported that indigenous patients lacked the ability to understand information they provided and could not make their own decisions about contraception. Additionally, some expressed overtly derogatory views including that indigenous women were dirty. Many reported withholding counseling on certain methods as a result of their indigenous patients' inability to properly use it. In contrast, a randomized control trial in Peru found no significant difference between the quality of family planning counseling provided to indigenous and nonindigenous ethnic profiles. However, the study had significant methodological limitations; the authors acknowledged that by using the same standardized patient to alternate ethnic profiles, they explored a relatively small range of ethnoracial characteristics.^37^ To our knowledge, no studies conducted in low-resource settings have examined family planning providers' implicit bias according to race or ethnicity. However, given the ubiquity of implicit racial and ethnic bias in health care,^14^^,^^38^ it would be surprising if Guatemalan family planning providers were immune to the cognitive trap that has befallen the thousands of physicians studied to date.

As such, further research is needed to document the extent of racial and ethnic bias in contraceptive care in low-resource settings. Initiatives to develop empiric evidence must accompany the growing conversation about widespread disrespectful and abusive reproductive health care globally.^39^ These efforts are especially important among indigenous people and those living in extreme poverty, who are among the most vulnerable.^40^^–^^43^ Indeed, given the strong negative correlation between indigeneity and both economic status and literacy in the national language, separating out the role of each individually will pose a significant methodologic challenge.

Initiatives to develop empiric evidence must accompany the growing conversation about widespread disrespectful and abusive reproductive health care globally.

## FINDING SOLUTIONS

Fortunately, there are a number of promising strategies to overcome racial and ethnic bias in health care delivery. Literature from the field of social-cognitive psychology suggest that providers can confront implicit racial bias once they are made aware of those biases through concrete activities and tests that elucidate unconscious biases and stereotypes.^44^^–^^46^ One study tested a package of interventions aimed at breaking racially prejudicial thought patterns using 5 cognitive exercises (Table) and showed a significant reduction in implicit bias.^46^ As Solo and Festin highlighted in their review, it is important to support providers in self-reflection rather than blame them.

**TABLE.** Habit-Breaking Exercises Shown To Decrease Implicit Bias Based on Race and Ethnicity^a^

Cognitive Strategy	Description
Stereotype replacement	Recognize your own responses that are based on stereotypes and teach yourself to have a different response
Counter-stereotypic imaging	Think of specific examples that do not fit with the racial and ethnic stereotypes you have been taught
Individuation	Overcome responses based on stereotypes by thinking about the person's individual qualities
Perspective taking	Imagine yourself in the place of the person from a racial or ethnic minority
Increasing opportunities for contact	Seek out chances to interact with members of other racial and ethnic groups

a **Adapted from Devine et al.** ^46^

Another method to confront racial and ethnic bias involves increasing providers' individualized interactions with members of other groups, for instance with colleagues of different racial or ethnic backgrounds,^45^ lending support to efforts to diversify health care provider workforces. It is important to acknowledge that individuals can hold bias against members of their own social groups, whether that bias is based on race and ethnicity or other client characteristics.

In addition to these person-level interventions, it is vital to recognize that the sources of racial and ethnic biases are deeply rooted in historical power structures and sociocultural forces that will likely require larger social movements to fully dismantle. Nonetheless, our organizational experiences and the current evidence on how to overcome biases leaves us optimistic that the global family planning community can successfully reduce its influence on clients' contraceptive choices.

Our organization offers family planning by training local nurses fluent in the indigenous languages spoken in the communities where they work. To expand our reach, we have developed an innovative partnership with the microfinance organization Friendship Bridge, which specializes in economic empowerment of primarily rural indigenous women in Guatemala, to provide a package of preventive health services, including a full range of family planning methods, to their clients. When we began to offer comprehensive contraceptive counseling and methods in 2014, we realized that family planning visits were often guided by providers' biases rather than by client preferences. For example, we witnessed our nurses encouraging women to initiate long-acting reversible contraception because they had “too many children.”

## SHARED DECISION MAKING

Our first step in confronting provider bias was to select a counseling approach to combat all types of provider bias. We implemented a shared decision making approach to contraception counseling, which prioritizes autonomy, control, and personal experiences in counseling by first clarifying a woman's unique preferences and guiding her to the “best fit” method (or no method at all). This model is well suited to combat provider bias of all types; indeed, a definition for provider bias quoted by Solo and Festin directly references “… failing to ascertain and respect the client's preference.”^47^ Moreover, shared decision making has been shown to improve patient satisfaction and increase continuation of chosen method, though it is only supported by rigorous evidence from high-resource contexts.^48^^,^^49^ We have had to consider our local cultural context to thoughtfully adapt this approach for the communities in which we work. Our efforts include working closely with Guatemalan providers (including some of the authors of this article) to create training materials that integrate realistic cases and vignettes based on actual, local client visits and use feedback from community nurses to improve and finalize training and counseling materials.

Our first step in confronting provider bias was to select a counseling approach to combat all types of provider bias.

The tenets of shared decision making may help providers become aware of their own biases. First, Solo and Festin discuss how emphasizing a client's right to make decisions could help make providers aware of their own biases. Similarly, the shared decision making approach encourages client-centered counseling in which the locus of control to make a final decision stands with the client. Second, Solo and Festin highlight that the negative impact of bias can in part stem from assumptions providers make based on bias as opposed to focusing on the individual client needs. Along these lines, shared decision making recognizes that each client is unique and encourages providers to personalize counseling by asking each client about her preferences rather than making assumptions. Finally, Solo and Festin discuss how the hierarchical medical model can further exacerbate bias. Shared decision making moderates this hierarchy by establishing client and provider as a team, in which the provider may be an expert on family planning methods, but the client is the expert on her personal situation and unique life circumstances.

Shared decision making establishes the client and provider as a team: the provider as an expert in family planning methods and the client as an expert on her personal situation and life circumstances.

Although a shared decision making approach has the potential to ameliorate bias in family planning, to effectively use shared decision making, providers must first confront their biases. A provider cannot successfully engage in this type of counseling if their biases prevent them from recognizing the validity of clients' preferences and desires. For example, consider the method-based bias held by many providers that the most efficacious methods—LARCs—are best. If providers are not aware of this bias or not able to move past this bias, they may have a hard time hearing, accepting, and responding to clients' other desires, such as using a method over which she has complete control. For these providers, it can be very difficult to offer clients other methods more aligned with the clients' personal preferences.

Two counseling tools widely available in low-resource settings have incorporated aspects of shared decision making. The World Health Organization's decision making tool^50^ and the Population Council's toolkit^51^ promote better provider-client interaction through decision algorithms that tailor counseling to each woman's individual circumstances. Each tool is accompanied by a core 1-day training curriculum to improve provider counseling skills, but these trainings dedicate little time to uncovering and addressing provider bias. However, similar to the other strategies reviewed by Solo and Festin, these existing shared decision making tools lack strong supporting evidence. Although both appear to improve the content of counseling—at least in the short-term—they have shown little to no impact on client use of contraception and lack rigorous evaluation of patient-centered outcomes, such as perception of respectful care.^52^^–^^56^ One possible explanation for these disappointing results is that counseling tools cannot truly achieve high-quality counseling using shared decision making without adequately unpacking provider bias.

Learning from these prior efforts, we have developed trainings on family planning counseling that specifically address provider bias before introducing shared decision making techniques and continue to reinforce the theme throughout the longitudinal curriculum. Although taking time to discuss overarching values and principles around client autonomy and nonjudgment can be important, we found that focusing on concrete examples has been more useful in encouraging awareness of personal biases. Lastly, we reinforce these themes through a rights-based framework, including training on reproductive aspects of Guatemalan human rights laws. We describe several strategies that we have found especially helpful (Box).

Counseling tools cannot achieve high-quality counseling using shared decision making without adequately unpacking provider bias.

BOX **Helpful Strategies To Increase Awareness of Provider Bias in Family Planning and Encourage Shared Decision Making**

Include training activities in which providers must identify their own preferences and make complex decisions in real-life scenarios outside of health care.

- For example, we ask nurses to choose a vacation destination and present information on a variety of factors, including cost, travel, time, and risk of crime, that impact this decision.
- These activities bring awareness to method-related biases by drawing parallels and reinforce the concept that there is rarely a single, best approach because each person has a unique context that will impact what is best for her.

Create realistic client case-based activities related to family planning, akin to an applied values clarification exercise.

- For example, we write cases to directly engage specific client-based biases including age, parity, and socioeconomic status.
- These activities bring awareness to client-based biases by eliciting providers' reactions to cases and helping providers to empathize with why clients may make decisions with which providers themselves do not agree.

Encourage health care provider group reflection on actual family planning visits that frontline workers have identified as being “difficult” for them.

- For example, we strive to recognize provider bias without judgment or blame to normalize this experience.
- These reflections encourage providers to explore which biases made those visits challenging.

Develop a longitudinal curriculum that includes not only follow-up trainings but also direct observation in the field.

- In our experience, classroom trainings alone are not enough to effectively confront provider bias.
- Direct observation of actual patient encounters reinforces recognition of biases and provides real-time feedback on counseling skills.

To date we have trained 20 providers in our curriculum. We would like to emphasize that while our approach is strongly based in theory, we are still in the early stages of implementation. Although we do not have enough longitudinal data to rigorously assess our program's impact on nurses' attitudes, patients' perception of provider bias, or clinical outcomes at this time, we hope to share our findings soon.

## CONCLUSION

We thank Solo and Festin for drawing attention to the issue of provider bias in family planning, a necessary step in ensuring client autonomy and satisfaction in this work in global health. We hope that our commentary encourages recognition of race and ethnicity as an important source of bias in family planning and that insights from our work may stimulate ideas for how to confront provider bias in other family planning programs.

## Notes

**En español**

**Perspectivas del prejuicio de los proveedores de servicios de salud en la planificación familiar desde una nueva iniciativa de consejería basada en toma de decisiones compartidas en comunidades indígenas rurales de Guatemala**

La raza, etnia, y el ser indígena deben considerarse como factores que contribuyen al prejuicio de los proveedores de servicios de planificación familiar a nivel mundial. Los esfuerzos para enfrentar el sesgo en la consejería de planificación familiar deben incluir estrategias concretas que promuevan el reconocimiento de los prejuicios, así como currículos longitudinales que permitan una retroalimentación sostenida y auto-reflexión.

**Peer Reviewed**

**Funding:** MN received funding for fieldwork from the Petersen Educational Enhancement Fund from the Brown University Office of Diversity and Multicultural Affairs as well as the American Women's Hospital Services Overseas Travel Assistance Grant from the American Medical Women's Association. The authors report funding from the Linked Foundation, American Medical Women's Association, Office of Diversity and Multicultural Affairs at the Warren Alpert Medical School at Brown University.
